# Effect of Triclosan and Silver Nanoparticles on DNA Damage Investigated with DNA-Based Biosensor

**DOI:** 10.3390/s22124332

**Published:** 2022-06-08

**Authors:** Jana Blaškovičová, Ján Labuda

**Affiliations:** Faculty of Chemical and Food Technology, Institute of Analytical Chemistry, Slovak University of Technology, Radlinského 9, 812 37 Bratislava, Slovakia; jan.labuda@stuba.sk

**Keywords:** DNA-based biosensor, silver nanoparticles, triclosan

## Abstract

Triclosan (TCS) is a broad-spectrum antimicrobial agent widely used in personal care, healthcare, and clinical practice. One of the most important aspects of toxicological profiling of compounds is their interaction with DNA. In human cells, TCS causes a significant reduction in DNA methylation. The involvement of TCS in chromosomal aberrations, DNA damage, and strand breaks, as well as DNA damage from TCS degradation products, was reported. AgNPs share similarities with TCS in terms of antimicrobial properties, enter the body after exposure, and are used even together with TCS in oral care products. Therefore, their mutual effect on the DNA is of interest. In this study, the electrochemical behavior of TCS on a glassy carbon electrode (GCE) and the biosensor with salmon sperm dsDNA (DNA/GCE), DNA damage by TCS present in phosphate buffer solution pH 7.4 and an additional effect of the immobilized AgNP layer on such DNA damage have been investigated. Two different sizes of AgNPs (about 15 and 37 nm) were tested. Using square-wave voltammetric signals of nucleobases, the portion of survived DNA was 64% in the presence of 15 nm AgNPs compared to 55% in its absence. The protective effect of AgNPs on DNA against TCS-induced DNA damage was found.

## 1. Introduction

Triclosan (TCS), 5-chloro-2-(2,4-dichlorophenoxy) phenol, is a broad-spectrum of antibacterial, antiviral, and antifungal agent frequently used as a preservative during the past 45 years in pharmaceuticals, personal care products, and cosmetics including toothpaste [1], soaps, shampoos, skin cleansers, detergents, deodorants, skin care lotions and creams [2], fabric and plastic additives [3,4], and impregnated in numerous different materials ranging from athletic clothing to food packaging [5]. TCS has also been used in surgical scrubs and in hand washing prior to surgery, to eradicate microorganisms such as methicillin-resistant Staphylococcus aureus (MRSA) [5]. In 1997, the FDA approved the use of TCS (0.3%) in Colgate Total toothpaste to prevent gingivitis and cavities. At the widespread use of TCS, it entered the environment and its presence in waters and waste waters in the US and Europe has been reported [2,6,7]. TCS has been detected in aquatic organisms, and sediments [8,9]. Significant levels of TCS are detected in body fluids in all age groups [5]. TCS has been detected in urine, plasma, serum and breast milk in humans all over the world [10,11,12]. TCS can accumulate in the human body, [13] where a large portion of free TCS is localized within the liver [14]. The prevalence of TCS exposure was indicated among youth [15].

Due to the bioaccumulation of TCS and its resistance to degradation, it represents a wide hazard to health. Hepatocellular adenomas and carcinomas were found in mice after exposure to TCS [16], additionally it enhanced liver fibrogenesis and tumorigenesis in mice [17]. Various toxic effects of TCS have been observed in studies affecting the nervous system [18,19,20], reproductive and developmental system [21,22], immune system, also at disruption of the endocrine system [23], thyroid hormone homeostasis [5], disturbance of the intestinal microbiota [24], antibiotic resistance [5,25,26], potential carcinogenicity [27], and neurotoxicity [28]. Recent data showed that TCS was associated with allergies and hay fever [29]. TCS can interfere with hormone regulation and fat metabolism that could cause hormone dyshomeostasis, induction of oxidative stress, apoptosis, and inflammation [30].

The widespread use of silver nanoparticles (AgNPs) in daily products shows great potential due to their effective biocidal activities [31,32]. AgNPs are the most widely used engineered nanoparticles in commercial products such as cosmetics, food, and medicine [33,34]. They share similarities with TCS in terms of antimicrobial properties, microbiome disruption, implications for antibiotic resistance, and effects on opportunistic pathogens [35]. At the same time, AgNPs as an alternative to TCS differ from TCS in structural properties, toxicity mechanisms, and bioavailability. AgNPs in toothpaste, mouthwashes [36], and other products such as impregnated toothbrushes and cosmetic products for the care of enamel remineralization and dental hypersensitivity could be responsible for inflammation of the gastrointestinal tract [37,38,39]. The nanoscale size range allows the entry of particles and directly affects intracellular structures [40]. Cellular uptake of engineered nanoparticles is often size dependent [41]. They persist in different organs up to several months. The accumulation of AgNPs decreases significantly with increasing size [42]. Furthermore, the retention of AgNPs was longer within a higher dose of smaller nanoparticles [43].

The antimicrobial effects of AgNPs depend on structural factors such as size, shape, coating, and ion release [44,45,46]. Biocompatibility and stability increases with a decrease in AgNPs size due to the higher surface-area-to-volume ratio [47,48,49]. The antibacterial activity of AgNPs smaller than 10 nm is mainly due to an Ag^+^ release [50]. The colloidal morphology has higher antibacterial activity when compared to other morphologies [51,52]. AgNPs-induced neurotoxicity could be reflected in irreversible degenerate spatial cognition [53,54]. A deep transdermal distribution of AgNPs was observed inside the body, followed by prolonged exposure [55]. However, even more alarming is the accumulation of TCS in body fluids by rapid absorption through the skin and gastrointestinal tract [56,57,58]. Therefore, concomitant exposure of the organism to TCS and AgNPs and their mutual effect on the DNA is of interest.

One of the most important aspects of the toxicological profiling of various compounds is their interaction with the DNA molecules [59]. TCS causes a significant reduction in DNA methylation in human cells [60]. An increase in dose-responsive chromosomal aberrations [16] and DNA damage [6,61] was observed with exposure to TCS but also to AgNPs [62]. The interaction of drugs and small species such as nanoparticles with DNA structure was investigated by electrochemical DNA-based biosensors, which became a very viable alternative due to high sensitivity in the detection of small differences in double helix structure compared to other methods [63,64]. Recently, the mechanisms of direct DNA damage caused by TCS degradation products was thoroughly investigated on a large scale of pH from 3.4 to 12.04 using a DNA-based biosensor [65]. At the interaction in solution of 0.1 M acetate buffer of pH 4.5; the condensation of double helix chain was found to lead to the difficulty of nitrogenous bases oxidation on the surface of the glassy carbon electrode (GCE) as well as the release of guanine moiety [65].

The novelty of our study is to contribute and characterize a prevention of DNA toward harmful chemicals such as TCS using another potentially protective DNA substance. Such an approach is known in the chemistry of immobilized species, including the construction of structured materials and biosensors. With respect to the known association of AgNPs with dsDNA [66], these nanoparticles of two different sizes, namely Ag1NPs (15 nm) and Ag2NPs (37 nm), were selected for this study. To prevent acidic or basic DNA denaturation, in this study the physiological pH value (0.1 mol·L^−1^ phosphate buffer solution at pH 7.4, PB) was used. The DNA damage by TCS was investigated using the DNA/GCE and AgNPs/DNA/GCE biosensors.

## 2. Materials and Methods

### 2.1. Materials

Triclosan (TCS), 5-chloro-2-(2,4-dichlorophenoxy)phenol, and low molecular weight salmon sperm double helix DNA were purchased from Sigma-Aldrich (Darmstadt, Germany). The 8.6 × 10^−4^ mol·L^−1^ stock solution of triclosan was prepared by dissolving TCS in ethanol: PB solution (1:3 *v/v*). The final concentration of TCS was achieved by diluting the stock solution with PB pH 7.4. DNA was dissolved in nanopure water to a concentration of 1 mg·mL^−1^. [Fe(CN)_6_]^3−/4−^, Na_2_HPO_4_ and NaH_2_PO_4_ were obtained from Lachema (Řečkovice, Czech Republic). AgNPs were prepared using a chemical synthesis protocol described by Martínez-Castañon et al. [67]. Other chemicals of analytical reagent grade purity were purchased from Mikrochem (Pezinok, Slovakia) or Lachema (Czech Republic). Nanopure water with a resistivity of about 18 MΩ·cm (Milipore Milli-Q system) was used for all experiments.

### 2.2. Apparatus

For all voltammetric experiments an Autolab PGSTAT12 potentiostat/galvanostat electrochemical system (Metrohm, Barendrecht, The Netherlands) driven by the software NOVA version 1.10.23 (Metrohm, Barendrecht, The Netherlands) was used. The three-electrode system consisted of a glassy carbon working electrode (GCE, Metrohm, Barendrecht, The Netherlands) with a disc diameter of 3 mm, Ag/AgCl/ 3 mol·L^−1^ KCl reference electrode and a platinum wire counter electrode (L-CHEM, Horka nad Moravou, Czech Republic). All measurements were performed in 20 mL glass cells at ambient temperature.

### 2.3. Preparation of the Biosensors

The GCE surface was mechanically cleaned on a polishing cloth (BUEHLER, London, UK) with 0.3 μm alumina suspension (Metrohm, Barendrecht, The Netherlands). GCE pretreatment was performed by polarization at a potential of 1.6 V for 300 s in 0.1 mol·L^−1^ PB solution of pH 7.4, and by stabilization of the CV response in 1 × 10^−3^ M [Fe(CN)_6_]^3−/4−^ redox indicator in cycling within the potential range from 1.0 to −0.8 V for 15 scans. The electrode modification was carried out by covering the pretreated GCE surface with 4 μL of DNA solution. After drying for 20 min., the DNA/GCE biosensor was stabilized by an incubation in 0.1 mol·L^−1^ PB solution for 2 min. For the preparation of Ag/DNA/GCE biosensor, 4 μL of 1 × 10^−3^ mol·L^−1^ AgNPs solution was dropped on the surface of the dry DNA/GCE biosensor and allowed to evaporate for 30 min. After 2 min. stabilization in PB solution, the Ag/DNA/GCE biosensor was used in the experiments.

### 2.4. Methods

#### 2.4.1. Cyclic Voltammetry (CV)

CV scans were recorded within a potential range from 1.2 V to 0.0 V at a scan rate of 100 mV·s^–1^ and a potential step of 2 mV in 0.1 mol·L^−1^ PB or 1 mmol·L^−1^ [Fe(CN)_6_]^3−/4−^ redox indicator in PB pH 7.4 with/without TCS in 20 mL electrochemical cell at laboratory temperature 21 °C.

#### 2.4.2. Square-Wave Voltammetry (SWV)

Square-wave voltammograms (SWV) were recorded under the following experimental conditions: potential step 4 mV, scan rate 200 mV·s^−1^, pulse amplitude 20 mV, frequency 50 Hz.

## 3. Results and Discussion

### 3.1. Characterization of the DNA/GCE Biosensor Stability

**Figure 1:**
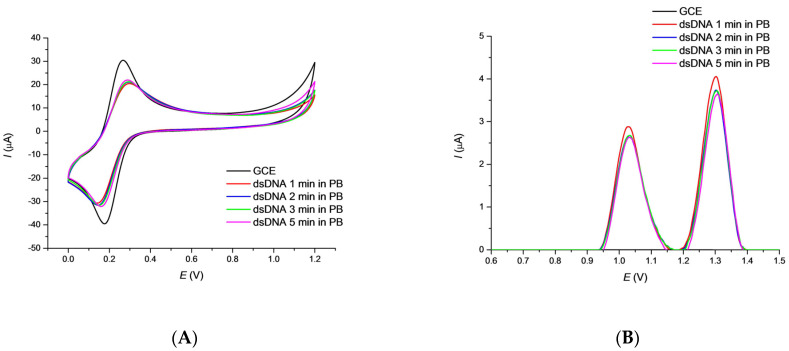
CV curves for 1 × 10^−3^ mol·L^−1^ redox indicator (**A**) and SWV (**B**) curves obtained for the bare GCE and DNA/GCE biosensor in 0.1 mol·L^−1^ PB pH 7.4 after treatment in the supporting electrolyte for a given time. Conditions: CV step potential 2 mV, scan rate 100 mV·s^−1^; SWV amplitude 20 mV, frequency 50 Hz, step potential 4 mV and scan rate 200 mV·s^−1^.

The first step in the preparation of the biosensor was the immobilization of DNA on the GCE working electrode, checked by the voltammetric response. A badly developed CV curve of the typical [Fe(CN)_6_]^3−/4−^ redox indicator on the DNA modified electrode, compared to the bare GCE is known to be the result of an electrostatic repulsion of indicator anions by the negatively charged surface-attached DNA backbone (Figure 1). Treatment of the newly prepared DNA/GCE biosensor in 1 × 10^−3^ mol·L^−1^ redox indicator in PB pH 7.4 for selected time periods has been used to to stabilize the DNA layer. The CV peak current values rise slightly with time of the biosensor incubation indicating an efficient and stable GCE coverage by DNA (Figure 1A).

Square wave voltammetry (SWV) is a suitable electroanalytical method for the determination of both the triclosan and the guanine (G) and adenine (A) moieties. The optimization study revealed (data not shown) that the set of pulse amplitude of 20 mV, frequency of 50 Hz, step potential of 4 mV, and scan rate of 200 mV·s^−1^ were best for monitoring. SWV peak currents of the G and A moieties decreased slightly within the given time intervals of the biosensor pretreatment in the supporting electrolyte (Figure 1B). As 2 min. treatment in PB solution was again the shortest time in which leaching of free DNA is nearly finished, it has been selected as appropriate for the newly prepared biosensor.

### 3.2. Effect of AgNPs on DNA/GCE Biosensor

**Figure 2:**
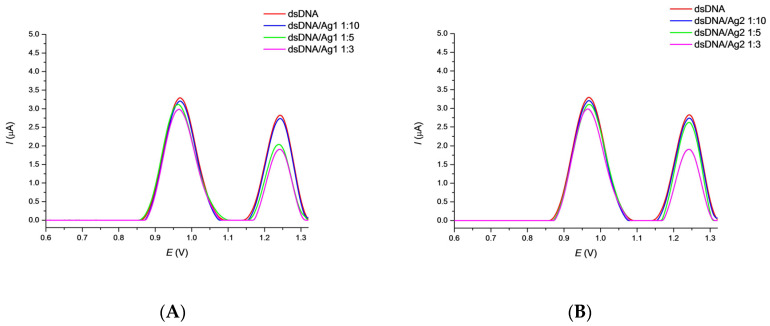
SWV curves recorded after 30 min. incubation of the DNA/GCE biosensor with Ag1 (**A**) and Ag2 (**B**) nanoparticles followed by 2 min. biosensor treatment in 0.1 mol·L^−1^ PB pH 7.4. SWV conditions: amplitude 20 mV, frequency 50 Hz, step potential 4 mV and scan rate 200 mV·s^−1^.

To avoid the signal interferences of AgNPs with DNA, an optimization study with several dilutions of nanoparticle solutions was performed by a set of voltammetric experiments. Stock solutions of the corresponding 1 × 10^−3^ mol·L^−1^ AgNPs were diluted 1:3, 1:5, and 1:10 (*v/v*) with PB pH 7.4 and dropped on the surface of DNA/GCE. After 30 min. incubation period and 2 min. biosensor treatment in 0.1 mol·L^−1^ PB pH 7.4, SWV curves were recorded (Figure 2). The anodic peak potential values of 0.975 V and 1.251 V, *E_p_*_G_ and *E_p_*_A_, were observed for the guanine (G) and adenine (A) moieties, respectively. The corresponding SWV current responses of the deoxynucleotides decreased with the concentration of nanoparticles applied. This decrease can be explained by a barrier effect for electron transfer evidently caused by an association of AgNPs with the dsDNA backbone as recently described [68]. Based on these results, the concentration of 1 × 10^−4^ mol·L^−1^ Ag1 and Ag2 nanoparticles was chosen for ongoing measurements which corresponds to a 1:10 *(v/v*) dilution of nanoparticles stock solution with PB. This AgNPs concentration should protect the DNA layer by association and, at the same time, not interfere with the detection of an effect of TCS in a further study. No GCE surface passivation by AgNPs was detected under these conditions.

### 3.3. Effect of TCS on DNA/GCE Biosensor

**Figure 3:**
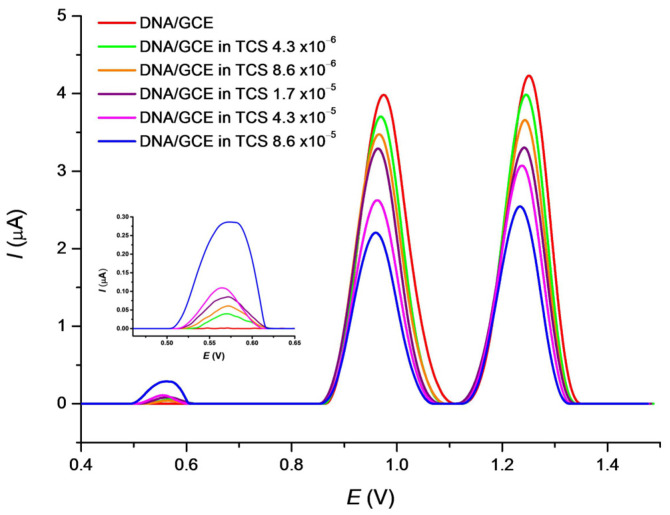
SWV curves recorded after 15 min. incubation of the DNA/GCE biosensor in TCS solutions of various concentration in 0.1 mol·L^−1^ PB pH 7.4. SWV conditions: amplitude 20 mV, frequency 50 Hz, step potential 4 mV and scan rate 200 mV·s^−1^.

The basic voltammetric behavior of TCS alone was previously described [65,69] and also confirmed in this work (data not shown). TCS exhibited the anodic response in the 0.57 V region vs. Ag/AgCl which depended on the TCS concentration within the range from 4 × 10^−6^ to 8 × 10^−5^ mol·L^−1^. However, TCS degrades the DNA molecule in concentration and time depending manner and our study was further directed at the detection of DNA changes. The effect of TCS on DNA was monitored using the SWV responses of nucleobase moieties after an incubation of the biosensor in TSC solutions for 15 min under stirring (Figure 3). Within the increasing concentrations of TCS, the *I_p_*_G_ values significantly decrease while the *E_p_*_G_ values slightly shift from 0.975 V toward the less positive potential of 0.960 V. The *I_p_*_A_ response also decreases and the *E_p_*_A_ values shift from 1.251 V to 1.233 V. Additionally, a SWV response of a TCS residue was detected as a small wide peak in the potential region of 0.57 V (Figure 3). This is in agreement with [65] where a similar peak was observed depending on the pH of the medium used. Changes in nucleobases responses confirm damage to DNA by the TCS drug, accompanied by a partial liberation of nucleobases bonds in the helix [70].

### 3.4. Effect of TCS on Ag/DNA/GCE Biosensor

**Figure 4:**
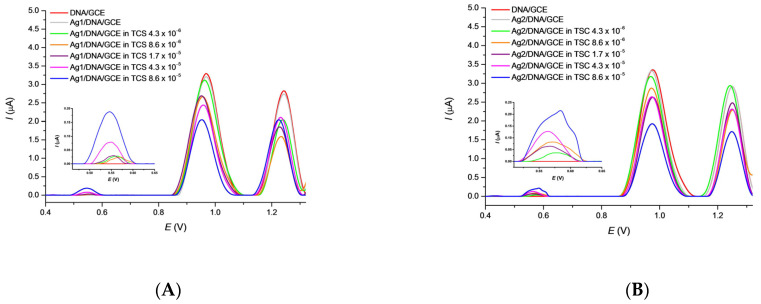
This SWV curves recorded after 15 min. incubation of the Ag1/DNA/GCE (**A**) and Ag2/DNA/GCE (**B**) biosensors in TCS solutions of various concentration in 0.1 mol·L^−1^ PB pH 7.4. SWV conditions: amplitude 20 mV, frequency 50 Hz, step potential 4 mV and scan rate 200 mV·s^−1^.

The mutual effect of TCS and AgNPs toward surface-attached DNA damage was tested using the Ag/DNA/GCE biosensor. The Ag1/DNA/GCE and Ag2/DNA/GCE biosensors were immersed in the TSC solution of various concentrations and allowed to incubate for 15 min under stirring. The SW voltammograms were then recorded immediately and directly in the TCS solution. In Figure 4A,B a decrease in the *I_p_*_G_ and *I_p_*_A_ values can be seen at both the Ag1/DNA/GCE and Ag2/DNA/GCE biosensors in proportion to increase in the triclosan concentration. The small *E_p_*_G_ shift to more negative potential values was observed only at Ag1/DNA/GCE. Again, the TCS residue can be observed as a slightly growing peak in the potential region of 0.57 V. The decrease in the *I_p_*_G_ response is less exhibited for Ag1/DNA/GCE. It is possible to conclude that both AgNPs used for the biosensor surface modification have protected the surface-attached DNA against its TCS-induced damage.

### 3.5. Protective Effect of AgNPs on DNA Damage by TCS

**Figure 5:**
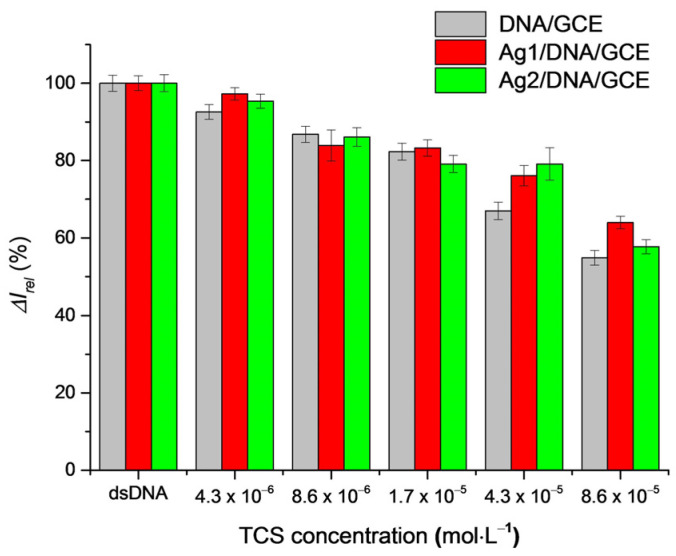
**The** amount of survived surface attached dsDNA expressed by its relative guanine moiety SWV peak current response in dependence on the concentration of TCS used at the 15 min incubation of DNA/GCE, Ag1/DNA/GCE and Ag2/DNA/GCE in TCS solutions.

An overall comparison of a decrease in the fraction of survived dsDNA (expressed by the relative current response of the nucleobases moieties) with an increase in the TCS concentration in the absence and in the presence of Ag nanoparticles is depicted in Figure 5. In most cases, a protection of DNA by the immobilized AgNPs (red and green columns) is seen.

The protective effect of AgNPs can be explained by their already reported direct interaction with the structure of dsDNA [68] and probably also by an interaction of TCS with AgNPs similarly to TiO_2_ nanoparticles [71]. It seems that Ag1NPs with smaller dimensions (15 nm) adsorb on the dsDNA structure in a greater amount than Ag2NPs (37 nm), and somewhat better prevent the interaction of triclosan with dsDNA. DNA protection could be further improved using a higher amount of the Ag nanoparticles (i.e., a smaller dilution of the AgNPs solution such as 1:5).

## 4. Conclusions

DNA—drug interactions are of permanent interest, particularly for species that indicate potential damaging effects such as triclosan. In this study, the experimental conditions for examining the effects of TCS on surface-attacheddsDNA were optimized and subsequently applied to the analysis of the DNA—TCS interaction with the electrochemical DNA/GCE biosensor. The negative effect of triclosan on DNA the helix structure was confirmed by monitoring the nucleobases responses. The silver nanoparticles immobilized over DNA exhibited some protection against TCS present in the solution phase, which revealed less damage to the DNA structure.

The results obtained here indicate a possibility of decreasing the toxic effect of TCS toward DNA by the presence of third species such as AgNPs. This can be of general interest in the case of a necessity of the elimination of unwanted effects of chemicals. For such further study we plan the investigation of morphology of the electrode surface modification as well as changes in the DNA structure using FTIR and Raman spectroscopies with special equipment allowing the measurement at the electrode body.

## Data Availability

Not applicable.
